# Imaging Analysis for Metastatic Risk Assessment in Adamantinoma: The Aid of Radiology in the Absence of a Histological Grading—An MRI-Based Risk Model Proposal

**DOI:** 10.3390/diagnostics15243124

**Published:** 2025-12-08

**Authors:** Mario Simonetti, Marco Colangeli, Paola Di Masi, Gabriele Bilancia, Valerio D’Agostino, Emanuela Palmerini, Gianmarco Tuzzato, Laura Campanacci, Alberto Righi, Amandine Crombé, Paolo Spinnato

**Affiliations:** 1 Diagnostic and Interventional Radiology, IRCCS Istituto Ortopedico Rizzoli, 40136 Bologna, Italygabriele.bilancia@gmail.com (G.B.); valerio.dagostino123@gmail.com (V.D.); 2 Department of Orthopaedic Oncology, IRCCS Istituto Ortopedico Rizzoli, 40136 Bologna, Italy; marco.colangeli@ior.it (M.C.);; 3 Osteoncology, Soft Tissue and Bone Sarcomas, Innovative Therapy Unit, IRCCS Istituto Ortopedico Rizzoli, 40136 Bologna, Italy; 4 Sylvester Comprehensive Cancer Center, University of Miami, Miami, FL 33124, USA; 5 Miller School of Medicine, University of Miami, Miami, FL 33124, USA; 6 Department of Pathology, IRCCS Istituto Ortopedico Rizzoli, 40316 Bologna, Italy; 7 Department of Musculoskeletal Imaging, Pellegrin University Hospital, 33076 Bordeaux, France

**Keywords:** bone neoplasms, bone sarcomas, magnetic resonance imaging, multidetector computed tomography, radiography, adamantinoma, sarcomas

## Abstract

**Background:** Adamantinoma is a very rare primary malignant bone tumor. A histopathological grading is still lacking, and as a result, metastatic risk stratification at diagnosis is challenging. Due to this, imaging could play a role in prognosis prediction and treatment strategy assessment. We aimed to evaluate baseline imaging features and their correlation with the development of metastatic disease. **Methods:** We retrospectively collected clinical (metastatic disease) and radiological data at baseline (Conventional Radiography, CT, MRI) of all consecutive patients with a histopathological diagnosis of adamantinoma at our sarcoma center between 2006 and 2022. Tumor location, dimensions, main radiological pattern (lytic, sclerotic, mixed), Lodwick–Madewell grading, periosteal reaction, multifocality, soft-tissue extraskeletal component, peritumoral edema, peritumoral enhancement, and vascular invasion were analyzed. Associations between the above-mentioned radiological features and metastatic disease at diagnosis or during follow-up were assessed. **Results:** Twenty-two patients were included (15 [68.2%] women, median age 27 years old, range 7–58 years old). Six out of twenty-two patients (27.3%) developed distant metastases (only two of them were dedifferentiated adamantinoma): two patients (9%) presented with metastatic disease at diagnosis, while four patients developed metastases during follow-up (18.2%). The following radiological features represent a significant risk for metastatic disease (*p* = 0.01): (i) presence of an extra-skeletal component (Odds Ratio [OR] = 75.40; 95% CI = 3.15–1802.71), (ii) vascular invasion (OR = 121.00; 95% CI = 4.28–3424.73), (iii) diffuse peritumoral edema (OR = 75.40; 95% CI = 3.15–1802.71), (iv) peritumoral enhancement (OR = 84.33; 95% CI = 2.93–2423.26). All other features analyzed were not significantly associated with the onset of distant metastases. Based on these above-mentioned MRI features, we built two risk models for metastatic disease (excluding peritumoral enhancement, which was not available in five patients, to be applicable on unenhanced MRIs): Model (A) = simultaneous presence of two of those three features (2/3) with a sensitivity of 100% (54.07–100%) and a specificity of 93.75% (69.67–99.84%). Model (B) = simultaneous presence of all three features (3/3) with a sensitivity of 83.33% (35.88–99.58%) and a specificity of 100% (74.1–100%). **Conclusions:** An accurate evaluation of baseline imaging studies (particularly MRI) in patients affected by adamantinoma may significantly aid in prognosis prediction and the selection of high-metastatic-risk patients. For these patients, strict follow-up controls and more aggressive treatments should be suggested after multidisciplinary discussions in sarcoma centers.

## 1. Introduction

Adamantinoma is an uncommon primary malignant tumor of the bone, accounting for about 0.1–0.5% of all primary bone tumors [1,2]. It is believed to arise from epithelial cells within the bone, though the exact pathogenesis remains uncertain. Numerous theories have been presented over the years regarding its histogenesis [3,4,5,6]; Fischer proposed congenital epithelial cell implantation in 1913 [3], naming it “primary adamantinoma of the tibia” due to the lesion’s remarkable histologic similarity to the jaw “adamantinoma” (ameloblastoma), whereas Ryrie [4] and Dockerty and Meyerding [5] hypothesized an origin caused by traumatic implantation.

The ancient Greek word “adamantinos” (extremely hard) is the source of the word “adamantinoma”. Generally, adamantinomas are slow-growing. These malignant bone tumors have a marked predilection for the diaphysis of tubular long bones (97% of cases, mainly the tibia and rarely the fibula), particularly at the mid-distal tibial shaft (80–85%) [1]. Satellite localizations of the main lesion at the level of the tibia should be sought at diagnosis, with ipsilateral fibula involvement present in a smaller percentage of cases (10–15%) [1]. This characteristic predilection for the tibia could be explained by some etiopathological theories, which suggest displacement and implantation of the skin basal epithelium during embryonic development, as in the leg the cartilage-derived bone is situated nearer the skin’s surface [1,7]. Clinically, it may remain silent for several years or generally manifest with nonspecific locoregional symptoms such as pain and swelling, associated or not with a soft tissue palpable mass [8,9].

**Figure 1:**
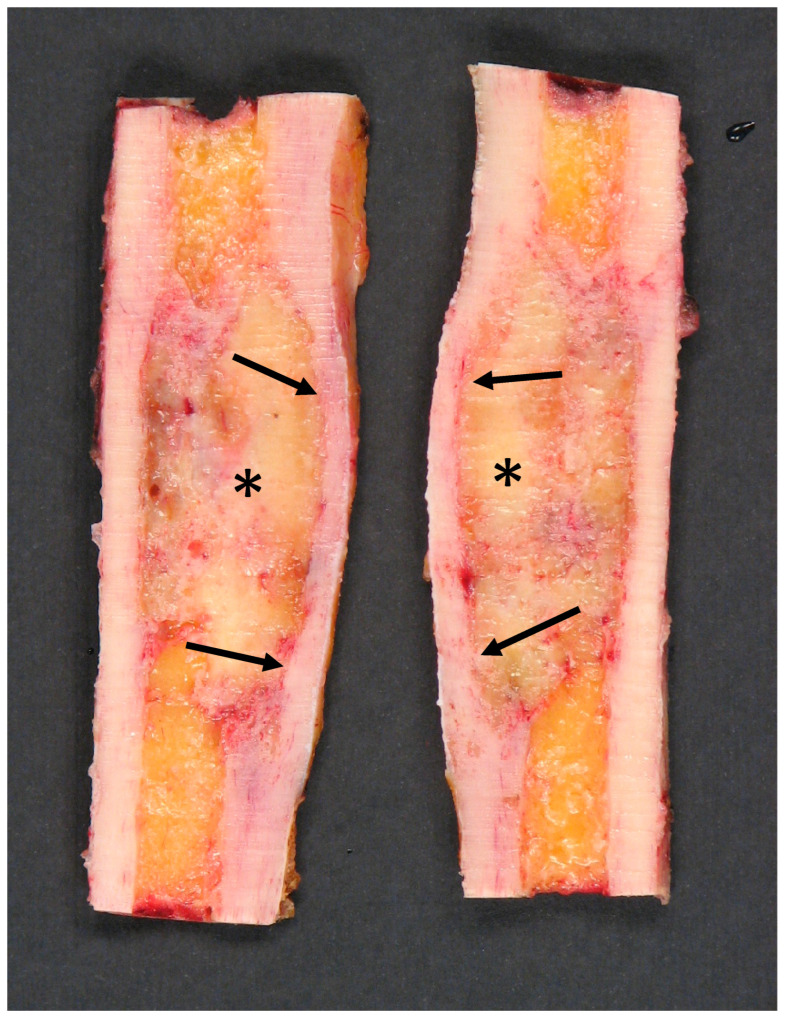
Bisected intercalar tibial resection specimen showing a fleshy intramedullary lesion (asterisks) that expanded the contour of the bone with diffuse cortical thinning/scalloping (arrows).

Macroscopically, adamantinoma usually appears as a fleshy intramedullary tumor that can expand the contours of the bone, and with cortical infiltration/thinning (Figure 1).

Histologically, adamantinoma is composed of nests or islands of malignant epithelial cells in an osteofibrous stroma. Different histological patterns of the epithelial component can be observed: basaloid (the most frequent), tubular, squamous, and spindle-shaped. Rarely, adamantinoma can show an epithelial-to-mesenchymal dedifferentiation into a high-grade sarcoma, which is called dedifferentiated adamantinoma [7,8].

**Figure 2:**
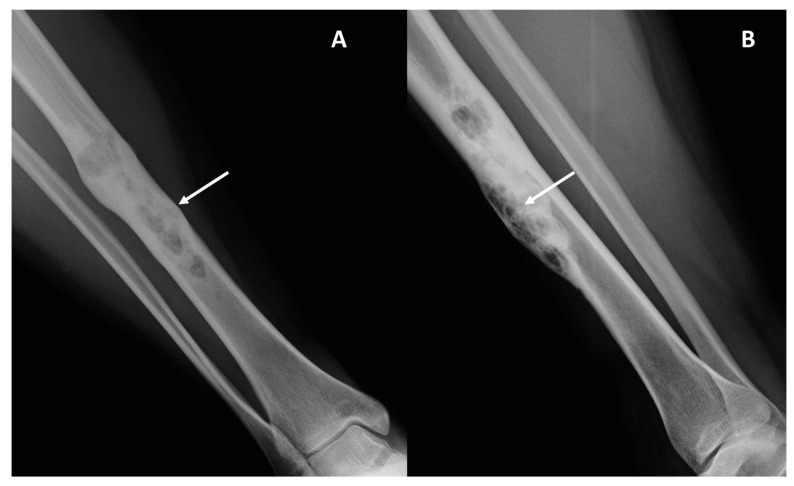
Conventional radiography (panel (**A**), antero-posterior view; panel (**B**), latero-lateral view) of the leg in a 38-year-old female with adamantinoma of the right tibial diaphysis. A mainly lytic geographic lesion with partial sclerotic borders and a “soap bubble” appearance (arrows) is displayed.

Radiographically, this tumor manifests as a mainly lytic lesion (possible area of sclerosis), often eccentric with cortical involvement, characterized by a geographic appearance with contextual radiolucent areas that give it a “soap bubble” appearance [7]. Single or multiple foci can be detected. The lesion can be circumscribed by a sclerotic margin and accompanied by various degrees of endosteal thinning. According to Lodwick–Madewell grading score, adamantinoma is usually classified as I (geographic) and A (sclerotic borders) or B (without sclerotic borders)—Figure 2 [10,11].

MRI is the preferred study for local assessment of the tumor. High signal intensity in both T1w and T2w sequences is described, along with homogeneous intense enhancement after contrast media injection [12]. No correlations between imaging features and histology or prognosis have been found so far [12].

Complete surgical resection with wide margins is the treatment of choice, as adamantinomas demonstrate resistance to chemotherapy and radiation. Prognosis is generally favorable following complete resection, though metastatic spread, particularly to the lungs, can occur (reported metastasis rate 12–45%) [9]. Conventional radiography is usually indicated for local follow-up after surgical removal and reconstructions, while chest CT scans (whole body CT or PET in high-risk patients) should be performed for distant follow-up controls [9,13].

To this date, there is no globally accepted histological grading, given its rarity and complex cell composition; thus, finding imaging biomarkers that correlate with the development of distant metastases and prognosis is paramount. Although considered a low-grade malignancy tumor, in a smaller number of cases it may show aggressive behavior, with a strong tendency for systemic spread leading to severe impairment of the patient’s outcome. Due to these different clinical behaviors and potential impact of therapeutic management and patient outcome, the aim of the study was to highlight clinical and imaging features associated with the occurrence of metastasis, which may suggest a more aggressive nature and a possible histologic dedifferentiation.

## 2. Materials and Methods

### 2.1. Study Design

This retrospective observational original research was approved by the ethics committee, and was conducted in accordance with the criteria set by the Declaration of Helsinki. For further details please see the ‘Institutional Review Board Statement’ section at the end of the manuscript.

Patients were initially identified through the pathological database of our Institution: histological diagnosis of adamantinoma. We retrospectively reviewed all the available data of patients with (i) histological diagnosis of adamantinoma, performed by expert pathologists from our sarcoma center between 2006 and 2022, (ii) adequate pre-operative local imaging including conventional radiography (CR) and one cross-sectional study (CT and/or MRI), and (iii) chest CT at initial disease staging and during further oncological controls with at least 2 years of follow-up.

All the imaging features collected and the main clinical data were matched with the onset of metastatic disease.

### 2.2. Clinical Data Collection

Clinical reports from our hospital were reviewed by a senior orthopedic oncology surgeon with 16 years of experience in the field (M.C.) and a radiologist (M.S.) with 4 years of expertise in musculoskeletal imaging.

The following clinical data were collected from the medical files using the computer software (RIS2020) available in our Institute: patients’ date of birth, date of diagnosis, age at diagnosis, sex, histological entity (including possible dedifferentiation), presence of symptoms at diagnosis (e.g., pain, swelling), and initial metastatic staging (baseline). Follow-up information was added: occurrence and location of metastasis and its date (number of months after diagnosis).

Finally, outcome data were collected to the latest available time point: patients’ status, categorized as non-evidence of disease (NED), alive with disease (AWD), or died of disease (DOD); and overall survival (OS) recorded in months, defined as the time between diagnosis and death related to illness or the patient’s last follow-up.

### 2.3. Imaging Acquisition Protocol

• Conventional radiography (CR) studies were performed with different equipment, with two orthogonal projections.
• CT studies were performed on different equipment and completed with bi-dimensional reconstruction (sagittal, coronal). Post-contrast scans were not consistently performed.
• MRI studies were performed on different equipment with high magnetic fields (1.5 Tesla or 3.0 Tesla system), using a standardized protocol that always comprised one T1-weighted imaging (WI), T2-WI with and without fat suppression (using Fat Sat, Short tau inversion recovery [STIR] or DIXON) and possibly at least one sequence of fat-suppressed T1-WI after contrast administration. At least two orthogonal acquisition planes were available for each patient.

### 2.4. Imaging Studies Review and Analyses

Two radiologists (P.S. and M.S.), with, respectively, 16 and 4 years of expertise in musculoskeletal imaging, reviewed in consensus all the available radiological studies (images and reports) for local and distant staging on a Picture Archiving and Communications System (PACS-Carestream Vue PACS v. 11.4.1.1102, Philips Healthcare, Amstelplein 2, 1096 BC Amsterdam, The Netherlands). In case of discordance, the results were discussed and re-evaluated with the addition of a third reader until reaching agreement (orthopedic oncologic surgeon with 16 years of experience M.C.).

The longest diameter in centimeters (LD, considering the three planes) was measured on each modality available (MRI or CT) at baseline, considering where the lesion was best visualized.

Based on the CR at baseline, the following data were assessed and recorded:

(1) Affected bone (e.g., tibia) and location (epiphysis, metaphysis, diaphysis, proximal, medial, distal).
(2) Periosteal reaction (present/absent, aggressive or non-aggressive, and type); please see specific references for extensive explanations [11].
(3) Skip lesions (present/absent, number, and site).
(4) Transition zone evaluation with Lodwick–Madewell grading (based on modified Lodwick–Madewell Grading System, 0–3) [10,11].
(5) Lesion main internal pattern (lytic, sclerotic, or mixed).

MRI imaging studies (or CT when MRI was not available) were analysed at baseline, and the following variables were recorded, when available:

(1) Tumor extra-skeletal extension in the soft tissues (categorized as: absent, mild [i.e., smaller than the intra-osseous component], moderate [i.e., equal to the intra-osseous component], severe [i.e., larger than the intra-osseous component].
(2) Relationship/contact with the near major vascular bundles (categorized as absent, near <3 mm, in contact, and vascular encasement), as already performed in previous sarcoma studies [14].
(3) Internal necrosis (categorized as absent or present [i.e., macroscopic non-enhancing areas after contrast media intravenous injection]).
(4) Peritumoral edema (categorized as absent, focal [only intraosseous, or only extra-osseous], and diffuse [intra- and extra-osseous]).
(5) Peritumoral enhancement (categorized as absent, focal, or diffuse).

### 2.5. Statistical Analysis

All statistical analyses were carried out using R Studio software version 4.2.0 (Vienna, Austria). A *p*-value of less than 0.05 was deemed significant. All tests conducted were two-tailed.

Fisher’s exact test was preferred over the Chi-square test to determine the statistical significance of the association between metastatic occurrence and categorical variables, considering the limited number of observations. Age was considered as a continuous variable and compared between groups using the Wilcoxon test. Associations between metastatic occurrence and numeric variables were assessed using an unpaired Student’s *t*-test or non-parametric Mann–Whitney test, depending on the Shapiro–Wilk normality test results.

For each extracted feature, the odds ratio (OR), along with 95% confidence interval (95% CI) and the corresponding *p*-value, was computed to estimate the risk of metastasis development associated with that feature.

The Kaplan–Meier method, with numerical and graphical results, was used to estimate metastasis-free survival in our cohort of patients.

For each binarized feature significantly associated with metastatic occurrence, sensitivity, specificity, and accuracy with 95% CI were estimated. Subsequently, a risk model for metastatic disease was also developed for the three variables that exhibited the highest correlation with metastasis development.

## 3. Results

### 3.1. Clinical Data

Twenty-two patients (15 women [68.2%]), with a median age of 27.0 years old (range: 7–58 years old), including 6/22 (27.3%) patients younger than 20 years old, 14/22 (63.6%) patients aged between 20 and 50 years old, and 2/22 (9.1%) older than 50 years old) with histopathological diagnosis of adamantinoma and available radiological studies were included in the study.

**Figure 3:**
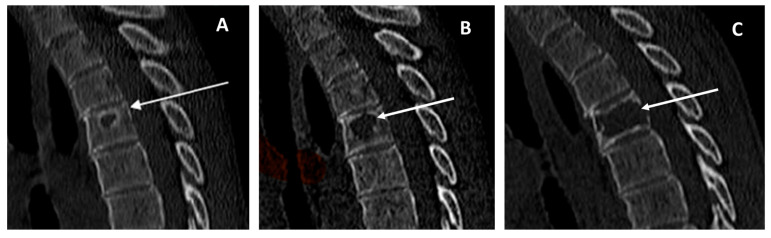
CT scans (sagittal view) in a female patient with a diagnosis of adamantinoma at the age of 26 years old. Chest CT-scan performed 8 months after diagnosis (panel (**A**)) revealed a geographic lytic lesion with sclerotic borders within the T4 vertebral body (arrow). The lesion was initially misdiagnosed due to the non-aggressive radiological appearance (Lodwick–Madewell grading 1A). The lytic lesion showed progressive dimensional increase over time in subsequent CT follow-ups (panels (**B**,**C**), arrows). A CT-guided biopsy revealed an adamantinoma metastasis.

**Figure 4:**
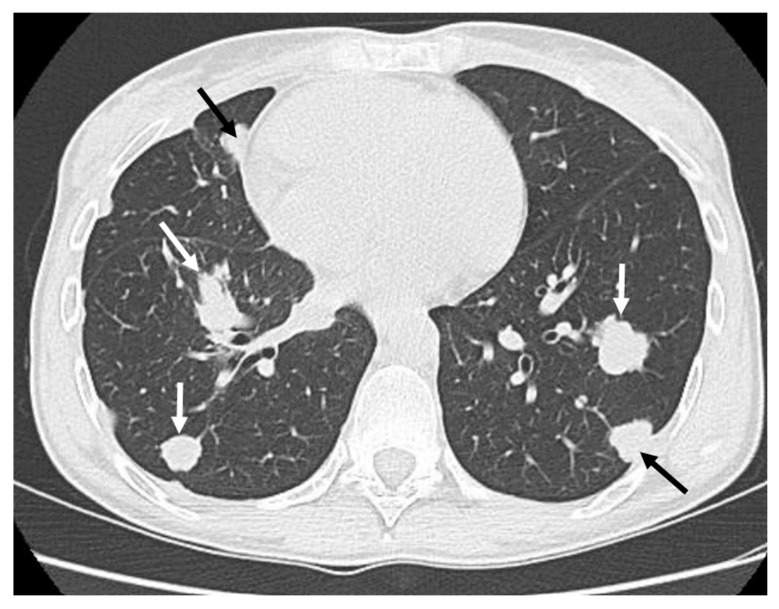
Classic appearance of bilateral “cannonball” lung metastases (white and black arrows) in a patient with adamantinoma.

Median follow-up time was 62 months (range: 8–180 months)—minimum follow-up for including patients in the study was 24 months (in patients who died before 24 months from diagnosis, follow-up was considered until the exitus). Overall, 6/22 (27.3%) developed distant metastases. Only two patients presented with distant metastases at diagnosis (9.1%), including one in the lungs and one in bones and lymph nodes distant from the primary site, while the other four patients developed metastases during follow-up controls (Figure 3 and Figure 4).

Initial therapeutic management was wide surgical excision in those without metastasis at diagnosis (20 patients), and surgical excision plus chemotherapy in patients with metastasis at baseline (2 patients). Among the remaining twenty patients free from metastatic disease at baseline, four (4/20–20%) developed metastases during follow-up controls, while sixteen did not develop metastases at all.

At the end of follow-up, 16/22 (72.7%) patients were classified as non-evidence of disease (NED), 2/22 (9.1%) patients were alive with disease (AWD), and 4/22 (18.2%) died of disease (DOD).

The patients’ main clinical and demographic data are summarized in Table 1.

None of the main clinical or pathological data significantly correlated with metastatic disease (Table 2).

### 3.2. Available Imaging Studies

In twenty-two patients (100%), at least one CR and MRI study was available before surgery. Five patients (5/22–22.7%) did not undergo a contrast medium injection during the MRI examinations.

### 3.3. Associations with Metastatic Occurrence

The following radiological features were significantly associated with metastatic occurrence:

An extra-skeletal extension (present in 100% [6/6] patients with metastasis versus 12.5% [2/16] without, *p* = 0.0004), vascular invasion (present in 83% [5/6] patients with metastasis versus 0% [0/16] without, *p* = 0.0002), diffuse peritumoral edema (present in 100% [6/6] patients with metastasis versus 12.5% [2/16] without, *p* = 0.0004), and peritumoral enhancement (present in 83% [5/6] patients with metastasis versus 12.5% [2/16] without, *p* = 0.001).

In Table 3, the relationships between imaging features and the occurrence of distant metastases are reported.

The odds ratios (OR) for the risk of metastatic disease of the above-mentioned statistically significant imaging features are reported in Table 4.

The OR univariate analysis (see Table 4) confirms a statistically significant association between several imaging characteristics, already highlighted by the Fisher’s exact test (see Table 3), and the risk of distant metastases. Specifically, we observed an OR > 1 (higher risk of metastasis) and *p*-values well below the significance threshold for extra-intraosseous extension (*p* = 0.01), vascular invasion (*p* = 0.01), peritumoral edema (*p* = 0.01), and peritumoral enhancement (*p* = 0.01). This confirms a relevant correlation between these imaging characteristics and the diagnosis of metastatic disease. These parameters prove to be key factors in assessing malignancy risk for patients.

**Figure 5:**
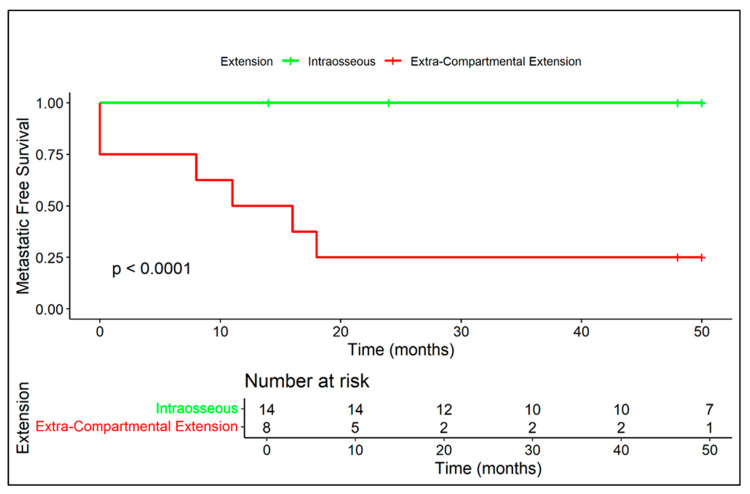
Extra-skeletal adamantinoma shows a markedly increased risk of early metastatic spread compared to the bone-confined form (median: 13.5 months vs. no events).

**Figure 6:**
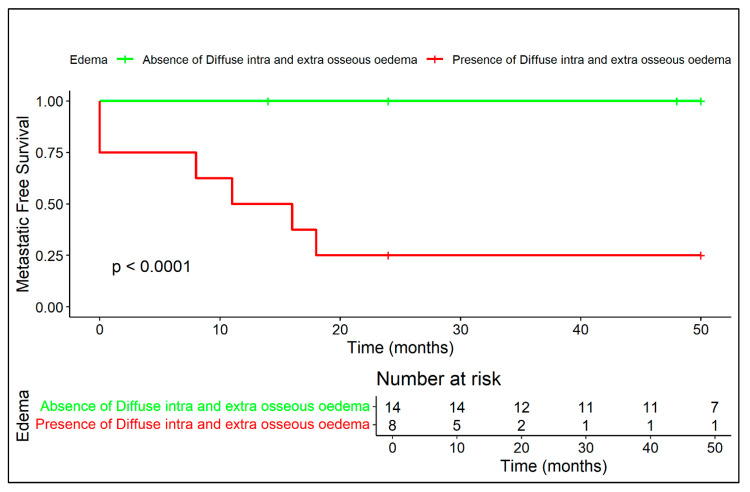
Diffuse peritumoral edema on MRI is associated with significantly reduced metastatic-free survival, confirming its role as a negative radiological prognostic factor.

**Figure 7:**
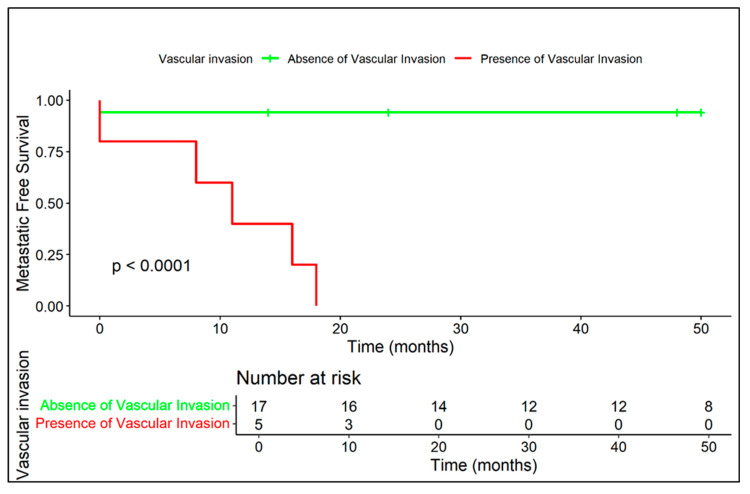
The presence of vascular invasion is strongly correlated with early metastasis (median: 11 months), whereas no vascular invasion corresponds to a highly favorable prognosis.

Kaplan–Meier curves related to metastatic-free survival are provided in regard to presence/absence of extra-skeletal component (Figure 5), diffuse peritumoral edema (Figure 6), and vascular invasion (Figure 7).

#### MRI-Based Risk Models

According to the above-mentioned results, we built two prognostic models considering the statistically significant imaging features (excluding the contrast-enhanced related one, i.e., peritumoral enhancement, which was not assessable in five patients). All these three features are optimally assessable via MRI studies.

In the performance analysis table (Table 5), we summarize the performance for two prognostic models based on two different sets of variables (2/3 features, Model A, and 3/3 features, Model B) in terms of sensitivity, specificity, and accuracy. The selected variables are those that exhibit the strongest correlation with the ‘metastatic disease’ variable and are identified as statistically significant based on the Fisher’s exact test and OR analysis: extraosseous component, vascular invasion, and peritumoral edema. Peritumoral enhancement was excluded, as mentioned before, because of missing data in five patients (absence of contrast media injection at baseline MRI). For each of these diagnostic features, one point was assigned in the prognostic models to reach the threshold (respectively, two and three features). Results of the risk models (A and B) are reported in Table 5.

For the **2/3** variables prognostic model (Model A), the sensitivity is 100% (with a confidence interval of 54.07% to 100%), indicating that the model correctly identifies all positive cases, but with a wide range of uncertainty. The specificity is 93.75% (95% CI: 69.67% to 99.84%), suggesting a high ability to correctly identify negative cases, but again, there is some variability in the estimates. These significant variabilities are attributed to the limited number of observations. The overall accuracy is 95.45% (95% CI: 77.16% to 99.88%), which demonstrates a strong overall performance, albeit with a broad confidence interval. The high variability is due to the limited number of observations.

For the **3/3** variables prognostic model (Model B), the sensitivity drops to 83.33% (95% CI: 35.88% to 99.58%), which still shows reliable performance, but with a wider confidence interval, suggesting more variability in detecting true positives. The specificity is 100% (95% CI: 74.1% to 100%), indicating perfect identification of negative cases. The overall accuracy remains the same at 95.45% (95% CI: 77.16% to 99.88%), reflecting impressive performance in general.

In conclusion, the 2/3 variables model shows higher sensitivity, while the 3/3 variables model achieves perfect specificity. Both models exhibit high accuracy, but the confidence intervals highlight some uncertainty, particularly in the sensitivity of the 3/3 model.

**Figure 8:**
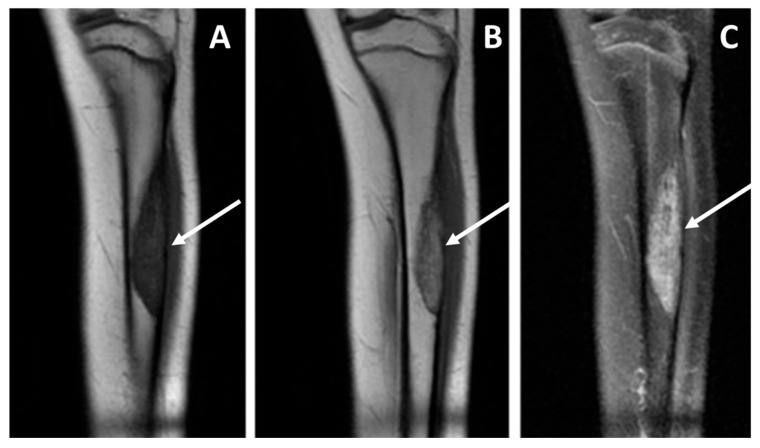
Baseline MRI study, coronal views, T1 weighted sequence (panel (**A**)), T1 weighted after contrast media injection (panel (**B**)), and fluid-sensitive sequence T2 weighted fat-sat (panel (**C**)), performed in a pediatric patient affected by adamantinoma. The study showed an intra-osseous lesion of the tibial diaphysis without a detectable extra-skeletal component (and consequently no vascular contact), and without any peritumoral edema. The patient did not develop metastatic disease; currently there is no evidence of disease more than 10 years after diagnosis.

**Figure 9:**
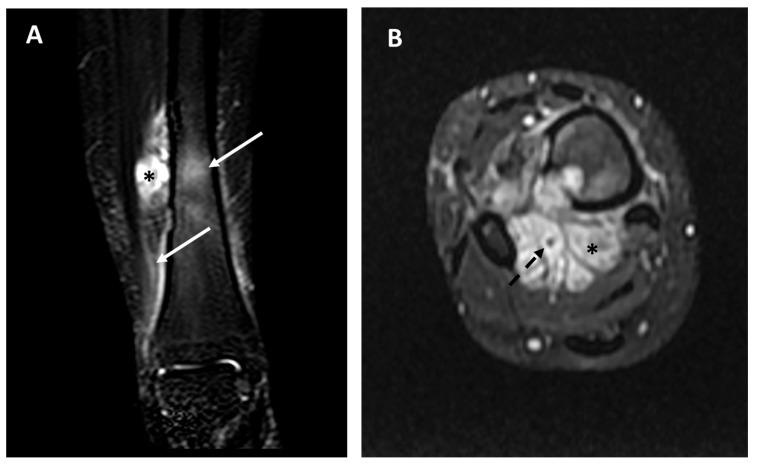
Baseline MRI study in a 26-year-old female affected by adamantinoma of the distal tibia; fluid-sensitive sequences (panel (**A**), STIR frontal view; panel (**B**), T2 weighted fat-sat axial view). Extraskeletal components (asterisks), diffuse peritumoral edema (arrows), and vascular encasement (dotted arrow) are well-detectable. This patient developed distant metastases 8 months after the diagnosis and died of disease.

In Figure 8 and Figure 9, we exemplify the application of the MRI-based prognostic models on two different patients.

**Figure 10:**
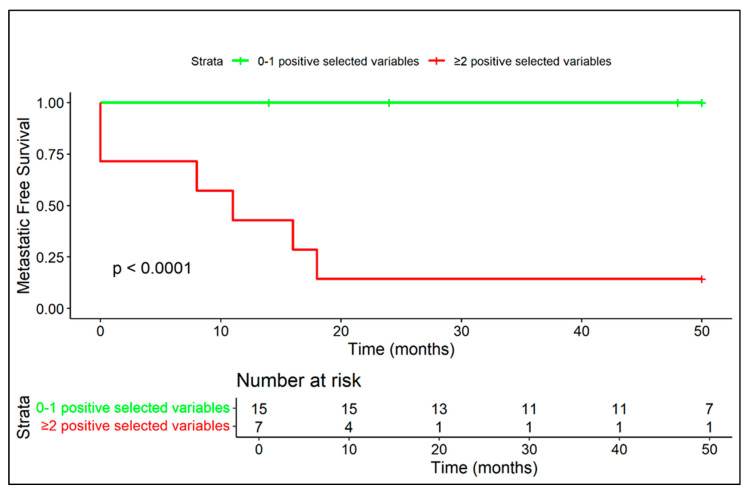
The coexistence of at least two negative prognostic MRI features (among diffuse peritumoral edema, extra-skeletal-component and vascular invasion) correlates with a high rate of metastasis and a median metastasis-free survival of 11 months.

**Figure 11:**
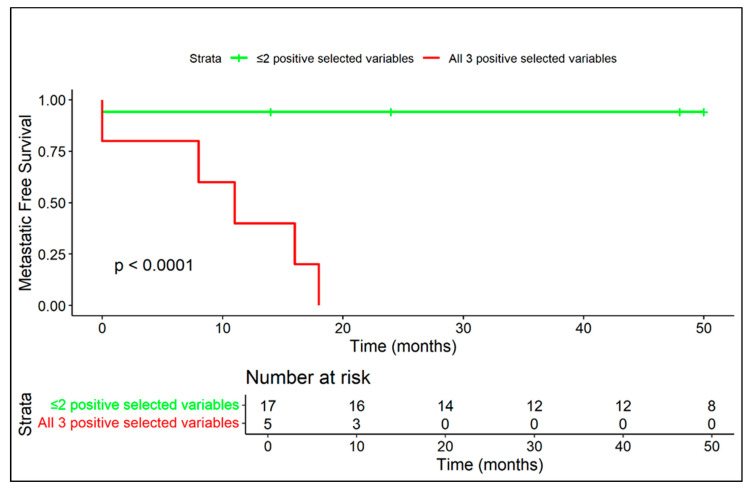
Patients with all three MRI variables (among diffuse peritumoral edema, extra-skeletal component and vascular invasion) exhibited extremely aggressive disease behavior, with early metastasis in all cases.

Kaplan–Meier curves related to metastatic-free survival applied to the two metastatic risk models are displayed in Figure 10 and Figure 11.

No associations were found between the evaluated clinical variables (age, sex, locations, and occurrence of pathological fractures) and metastatic disease.

## 4. Discussion

Adamantinoma is a rare bone cancer, accounting for about 0.1–0.5% of all bone cancers [1,2,15], that arises mainly at the level of the tibia, especially in young adults between the second and fifth decades of life. Although it is considered a slow-growing malignancy, the experience of our study based on 22 patients with histological diagnosis of adamantinoma showed that in six patients (~27.3%), this neoplasm had aggressive behavior and an unfavorable outcome for the patient. Histological diagnosis can be difficult in this type of neoplasm; therefore, radiological imaging can help the physician make a correct diagnosis. This neoplasm, which can mimic fibrous dysplasia (or other benign skeletal diseases), can easily cause diagnostic error and therefore be underestimated. In patients with a lytic, eccentric tibial lesion, in which the differential diagnosis also includes adamantinoma, a further diagnostic investigation by needle biopsy is recommended. Contrast-enhanced MRI may also be recommended in these patients, which allows for better characterization of the lesion and evaluation of tissue extensions close to each other, which in the case of adamantinoma has been shown to be an unfavorable prognostic factor for the patient. Particular attention should be paid to distant skeletal segments, even if a lesion may have characteristics of benignity (e.g., geographic lytic marginated lesion—Lodwick IA), in patients with adamantinoma. In fact, in a patient with bone metastases included in our study, the repetitive lesion appeared as a lytic lesion with edge sclerotic peripheral, specifically in a case of vertebral body involvement mimicking a Schmorl’s hernia (see Figure 3). Khanna et al. previously reported that adamantioma showed a larger size than osteofibrous dysplasia (OFD) and osteofibrous dysplasia-like adamantinoma [16]. In our study we included only patients with histologically confirmed adamantinoma. Another valuable aid for imaging analyses in clinical practice would be the distinction between adamantinoma and osteofibrous dysplasia (OFD), often presenting with similar radiologic features. Further research focused on this peculiar, and clinically relevant, differential diagnosis would be of interest.

In our study, with a prognostic aim, we did not find a statistically significant relation between the size of the lesion and the risk of metastasis, nor between the aggressiveness of the neoplasm and the patient’s prognosis, unlike many other sarcomas [17]. Some cases included in our study were particularly aggressive, including two patients with dedifferentiated adamantinoma, as expected [18,19,20], and, interestingly, even four patients without dedifferentiation at histological analysis.

Currently, the treatment of choice is block resection with large margins, associated with optional lymphadenectomy if lymph node involvement is suspected. Amputation as first-line treatment is not recommended, as it does not increase survival compared to more conservative surgical techniques [7]; it is instead the treatment of choice in cases of local recurrence or in those specific cases where en bloc resection of the tumor is not possible. Chemotherapy and radiotherapy have proven ineffective against this neoplasm and have not been shown to be generally effective as neoadjuvant therapy. Our study showed that among the imaging characteristics most correlated with the prognosis of the patient are extra-osseous extension of the neoplasm, the presence of edema, vascular invasion, and peritumoral enhancement, especially if they spread to both bone and soft tissues.

The study has some limitations that have to be discussed; first of all, its single-center design could hinder the generalizability of the results, although the extensive experience in a third-level reference center for musculoskeletal oncologic diseases should reassure us about the soundness of the study’s results. Furthermore, the small sample (22 patients), which is nearly inevitable when dealing with extremely rare diseases such as adamantinoma, particularly limits the statistical power of the research. As a matter of fact, to the best of our knowledge, this is the only radiological study in the literature about this condition in regard to prognosis prediction, being to this date often referred to in case reports, reviews, or simply descriptive analyses [12]. Finally, not all the patients included in our study were subjected to all the diagnostic modalities (with particular regard to missing contrast media sequences in several patients), which may lead to heterogeneous results in measurement and diagnostic features.

Metastatic disease can occur even in patients without histological dedifferentiation. Moreover, a histological grading is still lacking. Therefore, radiological imaging plays a pivotal role in metastatic risk stratification for patients diagnosed with adamantinoma. In this regard, based on the findings of this study, MRI represents a valid tool to suggest aggressive behavior of this neoplasm and a worse prognosis for the patient. This can affect the patient’s management strategy both from the point of view of follow-up and of processing; in particular, a closer and more extensive follow-up to the whole body can be suggested in patients with a greater risk of metastasis for early identification of repetitive lesions. From a therapeutic point of view, more aggressive, new/unusual, or neo/adjuvant chemotherapy to improve prognosis may be provided in these patients, even if future prospective research towards this aim is needed [21]. However, in that regard, further studies are needed, and given the rarity of the neoplasm, it may be desirable to consider multicenter collaboration.

## 5. Conclusions

Adamantinoma is a very rare primary tumor of the bone generally considered to be less aggressive. However, in our study, in more than a quarter of cases it showed highly aggressive behavior despite the presence/absence of histological dedifferentiation. MRI proved to be a clinically useful method for stratifying patients on the basis of the risk of metastasis, suggesting a worse prognosis in some patients. In particular, the degree of extra-skeletal soft tissue extension, vascular invasion, and peritumoral edema/enhancement were found to be imaging findings correlated with a poor prognosis. These data may suggest a different therapeutic care approach in patients diagnosed with adamantinoma based on baseline imaging studies already at baseline. However, further studies are needed to highlight the efficacy of a new possible personalized therapeutic strategy in these patients.

## Figures and Tables

**Table 1:** Main patients’ clinical data.

Age at Diagnosis (Sex)	Skeletal Location	Histological Dedifferentiation/ Dedifferentiated Adamantinoma	Metastasis at Diagnosis	Metastasis During Follow-Up	Radiotherapy (RT) and/or Chemotherapy (ChT)	Current Oncologic Status ^1^
26 (F)	Distal tibial diaphysis + Fibula	No	No	**Yes (8 months after diagnosis)—Bones, lungs**	RT + ChT	DOD
42 (F)	Proximal tibial diaphysis	No	No	No	No	NED
37 (F)	Proximal tibial diaphysis + Fibula	No	No	No	No	NED
14 (F)	Proximal tibial diaphysis	No	No	**Yes (11 months after diagnosis)—Lungs**	ChT	DOD
28 (F)	Mid-tibial diaphysis	No	No	No	No	NED
25 (M)	Proximal tibial diaphysis	No	No	No	No	NED
10 (F)	Proximal tibial diaphysis	No	No	No	No	NED
34 (M)	Mid-tibial diaphysis	No	No	No	No	NED
45 (M)	Mid-tibial diaphysis	No	No	No	No	NED
35 (F)	Proximal tibial diaphysis	No	No	No	No	NED
12 (F)	Mid-tibial diaphyisis + Fibula	No	No	No	No	NED
23 (M)	Distal tibial diaphysis	**Yes**	**Yes—Lungs**	**Yes—Lungs**	Cht	DOD
14 (F)	Distal tibial diaphysis	No	No	**Yes (18 months after diagnosis)—Lungs**	RT	AWD
7 (M)	Distal tibial diaphysis + Fibula	No	No	No	No	NED
20 (M)	Mid- and distal tibial diaphysis	No	No	**Yes (16 months after diagnosis)—Lungs**	ChT	AWD
22 (F)	Mid-tibial diaphysis	No	No	No	No	NED
42 (F)	Mid-tibial diaphysis	No	No	No	No	NED
28 (F)	Mid-tibial diaphysis + Fibula	No	No	No	No	NED
51 (F)	Mid- and proximal tibial diaphysis	**Yes**	**Yes—Bones, Lymph nodes**	**Yes—Bones, Lymph nodes, Lungs**	ChT	DOD
16 (M)	Mid-tibial diaphysis	No	No	No	No	NED
58 (F)	Proximal tibial diaphysis	No	No	No	No	NED
33 (F)	Distal tibial diaphysis	No	No	No	No	NED

^1^ NED = Non-evidence of disease, AWD = Alive with disease, DOD = Died of disease.

**Table 2:** Association between main clinical data and metastatic disease.

Variable	Statistical Test Performed	*p*-Value	Odds Ratio (95% CI)
Sex (M or F)	Fisher’s exact test	1.000	1.12 (0.11–8.5)
Histological dedifferentiation (Yes or No) *	Fisher’s exact test	0.505	4.46 (0.27–174.68)
Age at diagnosis	Wilcoxon rank-sum test	0.424	N/A

* Note: only two patients (2/22–9%) had de-differentiated adamantinoma, both with metastatic disease (100%). N/A: Not applicable.

**Table 3:** Association between imaging features and metastatic disease via Fisher’s exact test.

Imaging Feature	Non-Metastatic	Metastatic	*p*-Value
Radiological Pattern: Lytic (0), Mixed (1), Sclerotic (2)	Mixed (9), lytic (6), sclerotic (1)	Mixed (3), lytic (3)	0.69
Periosteal reaction: absent (0), non-aggressive (1), aggressive (2)	Absent (13), Non-aggressive (2), aggressive (1)	Absent (2), Non-aggressive (2), aggressive (2)	0.24
Lodwick type (I, II, IIIA, IIIB) *	II (1), III A (11), III B (4)	III A (5), III B (1)	>0.99
Extra-intra osseus extension: Intraosseous only (0), intra- and extra-osseous (1)	Intraosseous only (14), Extra-osseous (2)	Intraosseous only (0), extra-osseous (6)	0.0004 *
Vascular invasion: No vascular contact (0), Vascular contact or encasement (1)	No vascular invasion (16), Vascular invasion (0)	No vascular invasion (1), Vascular invasion (5)	0.0002 *
Unifocal (0), Multifocal (1)	Multifocal (6), Unifocal (10)	Multifocal (1), Unifocal (5)	0.62
Peritumoral edema: absent or focal (0), diffuse intra and extra-osseous (1)	Absent or focal (14), diffuse (2)	Absent or focal (0), diffuse (6)	0.0004 *
Peritumoral enhancement: absent (0), present (1)	Absent (11), Present (1)	Absent (0), present (5)	0.001 *
Longest diameter (cm)—cutoff 10 cm	>10 cm (7), <10 cm (11)	>10 cm (3), <10 cm (3)	0.67

* Statistically significant values in bold.

**Table 4:** Imaging features displayed (dichotomized) and metastatic disease risk assessment (Odds Ratio—OR), univariate analysis.

Imaging Feature	Non-Metastatic	Metastatic	Odds Ratio (95% CI)	*p*-Value
Extra-intra osseus extension: Intraosseous only (0), intra and extra-osseous (1)	Intraosseous only (14), Extra-osseous (2)	Intraosseous only (0), extra-osseous (6)	75.40 (3.15 to 1802.71)	**0.01** *
Vascular invasion: No vascular contact (0), Vascular contact or encasement (1)	No vascular invasion (16), Vascular invasion (0)	Novascular invasion (5), Vascular invasion (1)	121.00 (4.28 to 3424.73)	0.01 *
Peritumoral edema: absent or focal (0), diffuse intra and extra-osseous (1)	Absent or focal (14), diffuse (2)	Absent or focal (0), diffuse (6)	75.40 (3.15 to 1802.71)	**0.01** *
Peritumoral enhancement: absent (0), present (1)	Absent (11), Present (1)	Absent (0), present (5)	84.33 (2.93 to 2423.26)	0.01 *

* Statistically significant values in bold.

**Table 5:** Performance analysis for prognostic models based on selected imaging variables.

N. of Variables	Sensitivity (95% CI)	Specificity (95% CI)	Accuracy
**2/3**	**100% (54.07% to 100%)**	**93.75%** (69.67% to 99.84%)	**95.45%** (77.16% to 99.88%)
**3/3**	**83.33%** (35.88% to 99.58%)	**100%** (74.1% to 100%)	**95.45%** (77.16% to 99.88%)

## Data Availability

The original contributions presented in this study are included in the article. Further inquiries can be directed to the corresponding author.
